# Eco-friendly salt/alkali-free exhaustion dyeing of cotton fabric with reactive dyes

**DOI:** 10.1038/s41598-022-26875-8

**Published:** 2022-12-26

**Authors:** Tarek S. Aysha, Nahed S. Ahmed, Mervat S. El-Sedik, Yehya A. Youssef, Reda M. El-Shishtawy

**Affiliations:** Dyeing, Printing and Textile Auxiliaries Department, Textile Research and Technology Institute, National Research Centre, 33 EL Buhouth St., Dokki, Giza, 12622 Egypt

**Keywords:** Chemistry, Environmental chemistry, Organic chemistry, Process chemistry

## Abstract

The textile-wet process enormously consumes a large volume of water and chemicals, and thus awareness of cleaner production has been growing to protect the environment from the industrial effluents. In this context, reactive dyeing of cellulosic materials such as cotton fabrics is a major sector of textile coloration that necessitates the use of a large amount of sodium sulfate or sodium chloride and alkali to exhaust and fix the dye molecules with cellulosic macromolecules, respectively. However, the remaining salt and alkali in the effluent badly affect the environment. For this purpose, the use of trisodium nitrilotriacetate (TNA) in reactive dyeing of cotton fabrics was hypothesized to have a double benefit, one as an exhausting agent (organic salt) and the second as a fixing agent (organic base). Thus, the exhaust dyeing characteristics of cotton fabrics using C.I. Reactive Yellow 145 (RY145) was optimized under different conditions of TNA concentration, alkali concentration, temperature, and dyeing time. The color strength and the primary and secondary exhaustion values were also investigated with an eye on those values obtained using the conventional dyeing method. The characterization of effluent samples with RY 145 taken after dyeing using TNA compared with conventional dyeing indicated an efficient reduction of COD, BOD, and TDS values by 99, 97, and 97%, respectively. The new dyeing method was implemented using C.I. Reactive Black 5 (RB5), C.I. Reactive Blue 160 (RB160), and C.I. Reactive Red 24 (RR24) to reveal good dyeability and fastness properties comparable with those obtained using the conventional method. The overall results obtained suggest the suitability of TNA as an environmentally friendly agent suitable as an exhausting and fixing agent of cellulosic fabrics.

## Introduction

Cellulosic fibers are of main crops that are widely exploited in textile industries. Textile fabrics made from such fibers are superior in comfort and environmentally friendly. Coloration of cellulosic fabrics relies mainly on the excellent dyeing characteristics obtained from reactive dyes. This class of dyes gets fixed into the fabric by forming covalent bonds via nucleophilic substitution or nucleophilic addition of the dye molecules with the hydroxyl groups of cellulose under alkaline conditions^1–3^. However, cellulosic fabrics in water acquired negative surface charges ^4^ that necessitate using a large amount of inorganic salt (i.e. sodium chloride or sodium sulphate) to neutralize the surface charges and promote dye exhaustion from the dye bath into the fabrics. Moreover, the use of inorganic alkali (i.e. sodium carbonate) to accomplish the dyeing process with satisfactory levels of dye-fiber bond fixation with improved wet fastness properties is needed.

The required amounts of inorganic salts and alkali are necessary to improve the reactive dye exhaustion and dye–fiber fixation efficiency^5–9^. The electrolyte quantities can be as high as 100 g/L depending on the shade required, dye structure and the dyeing method ^10^. Generally, the reactive dyeing process consumes a noticeable quantity of water and almost all inorganic electrolytes, alkalis and unfixed dyes are discharged to the dyeing effluents, which are in most cases, polluted water and soils^11–14^. In this context, the exhaust dyeing process that represents the most widely used process of dyeing cotton fabrics with reactive dyes could lead to a substantial environmental impact with higher consumption of water and chemicals as well as the emission of dye effluents ^14,15^. Thus, the performance evaluation of a modern reactive dyeing technology and process optimization has become necessary to reduce chemicals, energy and water consumption^16–18^. Attempts to reduce the amounts of inorganic electrolytes on reactive dyeing have included the application of the bifunctional reactive dyes, which can react more readily with cellulose and showing better dyeing performance even at low salt and alkali concentrations.

There is also a growing interest in using innovative, eco-friendly, and sustainable reactive dyeing procedures accomplished by the use of biodegradable alternatives to the non-biodegradable inorganic salts and/or alkali^19–22^. Previously, we have reported the viability of using an organic amine salt namely, ethylene diamine tetraacetatete tetrasodium salt (also known as sodium edate, SE) as an alternative bio-degradable alkaline salt for the exhaust reactive dyeing of cotton fabric and its blends to the conventional inorganic salt and alkali^23–25^. This salt also proved to be possible alternative to urea/alkali in reactive printing past through steaming process ^26^. Also, Prabu and Sundrarajan ^27^ have reported the use of trisodium citrate salt as an alternative to conventional inorganic electrolytes for exhaust dyeing of cotton with reactive, direct and solubilised vat dyes. In addition, the use of trisodium nitrilotriacetate (TNA) and tetrasodium *N*,*N*-biscarboxylatomethyl-l-glutamate (GLDA) proved as an alternative in pad-steam dyeing process of cotton with reactive dyes ^11,28–30^.

In continuation of research interest toward cleaner production in textile industries the use of trisodium nitrilotriacetate (TNA) as an organic salt and alkali (pH 11, 1% aqueous solution) in batch dyeing of cotton with reactive dyes was studied^11^. The effects of TNA concentration on the dyeing properties of different reactive dyes containing MCT, VS and MCT/VS groups on the exhaust dyeing process of cotton fabrics, the dye exhaustion and fixation were investigated. CIELab colour coordinates and colour strength in terms of K/S values were determined and compared to the conventional reactive dyeing on cotton fabrics. Several variables, including the reactive dye type and the amounts of salt/alkali used for all the reactive dyes were investigated. The TNA-reactive dyeing results and dye bath effluents were compared with those obtained by the conventional processes. The fastness properties of dyed cotton fabrics using different reactive dyes were also evaluated.

## Materials and methods

### Materials

**Figure 1:**
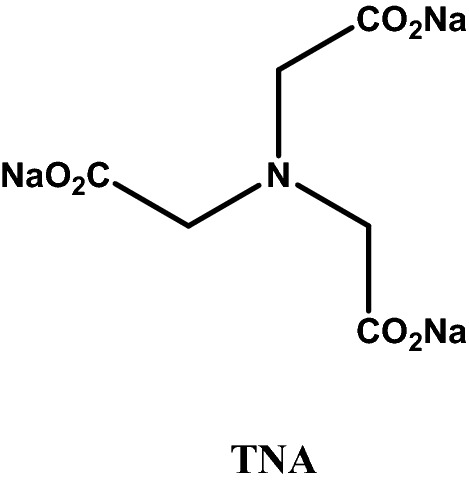
The chemical structure of trisodium nitrilotriacetate (TNA).

A bleached cotton fabric in plain weave (160 g/m^2^, Ne 120/2, 69 End/cm, 22 Picks/cm) supplied from El-Mahalla El-Kobra Company, Egypt, was scoured by boiling in a bath containing 5 g/L sodium carbonate and 2 g/L nonionic detergent (Sera wash M-RK DyStar, Egypt) for 3 h, then rinsed with cold water and air-dried at ambient temperature. Four commercial reactive dyes, comprising one hetero-bifunctional monochloro-s-triazine/vinylsulphone (MCT/VS) dye (C.I. Reactive Yellow 145), one homo-bifunctional Bis(MCT) dye (C.I. Reactive Blue 160), one homo-bifunctional Bis(VS) dye (C.I. Reactive Black 5) and one monofunctional MCT dye (C.I. Reactive Red 24) were used in this investigation. These dyes were supplied by DyStar and Oh Young Industrial Co. Ltd., and used as received. The C.I. generic name and chemical structures of these dyes are illustrated in Table 1. All the obtained dyestuff were of commercial quality and used without further purification. Sodium sulphate anhydrous (SS), sodium carbonate (SC) and trisodium nitrilotriacetate (TNA) Fig. 1 was purchased from Fluka, Germany.

**Table 1:** Properties of the reactive dyes and its chemical structures.

CI generic name	Commercial name	λ_max_ (nm)	Dye structure	Dye class M. wt.
CI Reactive Yellow 145 (RY 145)	Remazol Yellow 3RS	420		MCT/VS 1026.22
CI Reactive Black 5 (RB 5)	Remazol Black B	598		Bis (VS) 991.79
CI Reactive Red 24 (RR 24)	Jakofix Brilliant Red HB	535		MCT 774.03
CI Reactive Blue 160 (RB 160)	Suncion Blue H-ERD (Oh Young)	618		Bis (MCT) 1309.83

### Dyeing methods

The viability of using TNA-method in reactive dyeing was studied, corresponding the optimum dyeing temperature/time during the exhaustion and fixation stages by varying the time from 0 to 60 min and temperature from 40 to 80 °C. The effect of adding TNA salt instead of the conventionally sodium sulphate and sodium carbonate was investigated at the same dyeing conditions.

**Figure 2:**
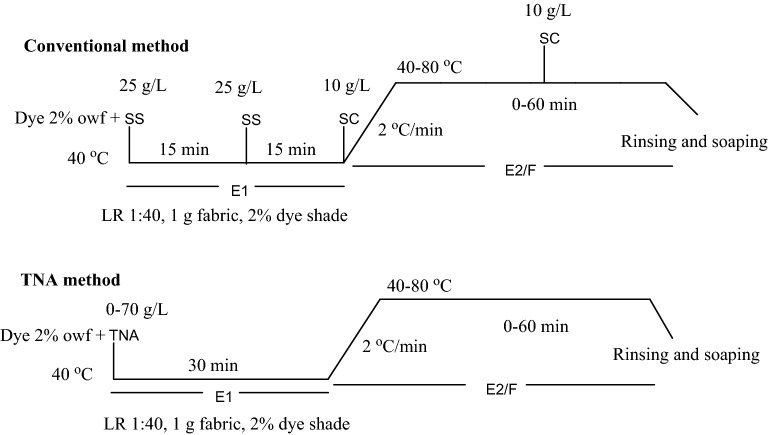
The dyeing profile of conventional and TNA methods.

A series of SS/SC-free TNA dyeings was produced using 2% shade of the dye at a liquor ratio of 1:40. The dyeing process was started at 40 °C with various amounts of TNA (0–70 g/L) were added for 30 min primary exhaustion time, unless otherwise specified. The dyeing process continued for further 60 min, while the temperature was then raised to 40–80 °C, unless otherwise specified, to complete the secondary exhaustion and fixation stage. The SS/SC conventional dyeing of cotton fabric (reference sample) was carried by replacing trisodium nitrilotriacetate (TNA) with sodium sulfate (SS) 50 g/L and sodium carbonate (SC) 20 g/L. SS was added at 40 °C in two portions within 30 min, SC was added in two portions within 1 h. All the dyed fabric were rinsed with cold water, and the unfixed dye was washed using a solution of 2 g/L sodium carbonate and 2 g/L non-ionic detergent at LR 1:50 and boiling for 30 min (Fig. 2).

The K/S, exhaustion and fixation of the dyed sample using TNA were compared with conventional dyed sample using SS 50 g/L and SC 20 g/L. The color strength (K /S) and the color coordinates of all dyed fabrics were expressed in the CIELAB color space system (often denoted as L*, a*, b* coordinates). From which the value of L* represent lightness or darkness of the sample (a higher lightness value represents a lower color yield); a* denote redness if positive value or greenness if negative; b* represent yellowness if positive or blueness if negative and C* specifies chroma and h denotes hue angle were also measured using a Hunter Lab UltraScan PRO spectrophotometer (USA) under illuminant D65, 10 standard observer.

The total color difference values (ΔE*) between the TNA dyed sample and the conventional one was calculated using the Eq. (1):

1 $$\Delta \mathrm{E}*=\sqrt{({\Delta \mathrm{L}*)}^{2}+{\left(\Delta \mathrm{a}*\right)}^{2}+{\left(\Delta \mathrm{b}*\right)}^{2}},$$

where ΔL^*^, Δa^*^ and Δb^*^ are the differences of L^*^, a^*^, b^*^ color parameters corresponding TNA and conventionally dyed samples, respectively.

The color strength (K/S) of dyed fabrics after washing was recorded using the technique of light reflectance by applying Kubelka–Munk Eq. (2) ^31^.

The reflectance (R) of the dyed fabrics was measured on Shimadzu UV2401 spectrophotometer (Japan).

2 $$K/S = \frac{{\left( {1 - R} \right)^{2} }}{2R},$$

where R = Decimal fraction of the reflection of the dyed fabric, K = Absorption coefficient, and S = scattering coefficient.

The absorption spectra of the dye solutions before and after dyeing was recorded by a Shimadzu UV2401PC UV–Visible spectrophotometer at the value using a calibration curve previously obtained using known dye concentrations (g/L) in water for calculating the % of exhaustion and fixation of dyes onto cotton fabrics.

The extent of exhaustion achieved for 2% (owf) dyeing on cotton fibers was determined using spectroscopic analysis of the dyebath before and after dyeing at different times. The calibration curve for each dye was determined by measuring the absorbance of the dye solution of known concentration. The percentage of the dyebath exhaustion achieved for each dye was calculated from the Eq. (3).

3 $$\% E =\frac{{A}_{1}-{A}_{2}}{{A}_{1}}\times 100,$$

where A_1_ is the concentration of the dyebath before dyeing, and A_2_ is the concentration of the dyebath after the neutral exhaustion stage (primary exhaustion, E_1_) and/or the alkaline stage (secondary exhaustion, E_2_).

The determination of the dye fixation ratio (%F) was measured by stripping the dyed samples at boiling for 30 min (liquor ratio 1:50) in a boiling bath containing 2 g/L sodium carbonate SC and 2 g/L nonionic detergent until all unfixed dyed was removed. The dye fixation ratio (%F) was calculated as presented in Eq. (4).

4 $$\% F =\frac{{A}_{1}-{A}_{2}-{A}_{3}}{{A}_{1}-{A}_{2}}\times 100,$$

where A_3_, the concentration of dye extracted after boiling using a solution of 2 g/L sodium carbonate and 2 g/L non-ionic detergent for 30 min at boiling LR 1:50.

### COD, BOD, TDS and TSS measurements

Laboratory analyses of the chemical oxygen demand COD, biochemical oxygen demand BOD and the total dissolved salt TDS of the residual dyebath was carried out in accordance with Standard Methods for Examination of Water and Wastewater [APHA, American Public Health Association Standard Methods for the Examination of Water and Wastewater, 23ed edition, Washington, D.C (2015)].

### Fastness testing

After washing-off using 2 g/L SC and 2 g/L nonionic detergent until all unfixed dyed was removed, a specimen of dyed cotton fabrics of 2% owf depth of shade were tested according to ISO standard test methods. The wash fastness test was assessed in accordance with the standard method ISO 105-C06 B2S (2010) using 4 g/L of ECE detergent, 1 g/L of sodium perborate, 25 steel balls) at 50 °C for 30 min and at a liquor ratio of 50:1. Fastness to acidic and alkaline perspiration was determined with a perspirometer set at specific pressure, temperature and time in accordance with ISO 105-E04 (2008). Any change in colour of the dyed samples (Alt) and colour staining on the adjacent undyed cotton (SC) and polyester (SP) fabrics was then assessed with the corresponding ISO grey scales for colour change and staining rates. Light fastness was also assessed using a Xenon arc lamp test in accordance with ISO 105-B02 (2013).

## Results and discussion

**Figure 3:**
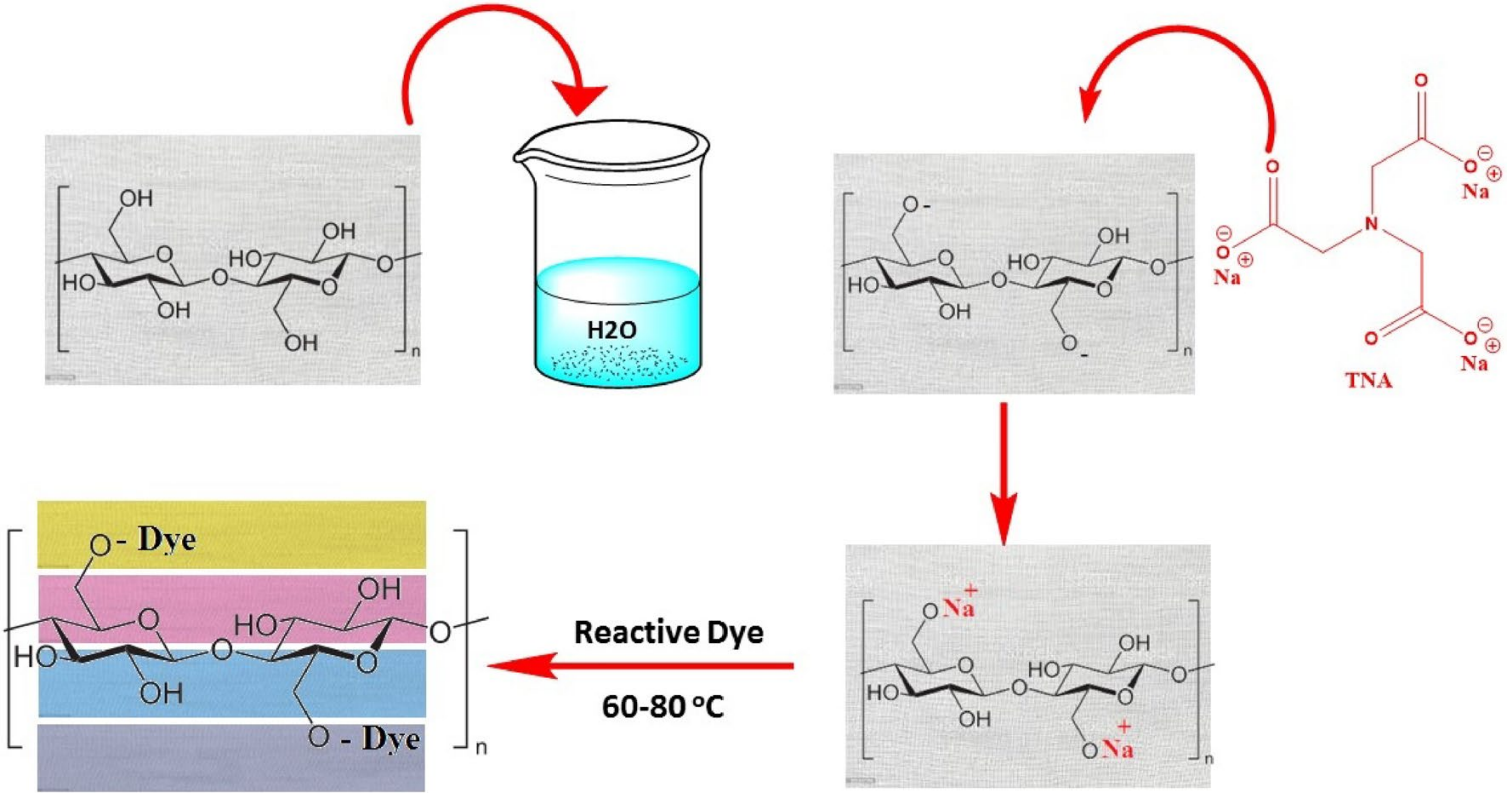
Schematic diagram of the interaction of TNA salt with cotton fabric during exhaustion dyeing with reactive dye.

The present study explores the use of TNA (Fig. 1), being an organic sodium salt, it can act as an electrolyte for exhausting anionic reactive dyes onto cotton fibers and promote ultimately dye fixation and color yield because of its alkalinity, like sodium editate ^24–26^. The dyeing interaction of TNA salt for exhaustion dyeing method with reactive dye is presented in Fig. 3.

It is known that the dyeing being an exothermic process, the exhaustion of the given reactive dye is anticipated to be of lower values at higher dyeing temperature. Furthermore, the problems associated with reactive dye hydrolysis and low dye uptake would be less pronounced if the reactive dye used is more stable against hydrolysis and more robust for variation in the dyeing conditions. Since the use of an organic base would be expected to be more convenient for dyeing cotton fabrics with reactive dyes, therefore, the purpose of this work was to examine the dyeing performance of reactive dye class variation using TNA. The pH of dyebath containing 50 g/L of TNA is about 10 activating the nucleophilic substitution reaction of the reactive group located in the dye molecule with the primary hydroxyl group in the cotton fabric, similar to sodium edate behavior ^11,23–25,28^. The following investigation study optimized the use of TNA as an exhausting and fixing agent for cotton dyeing using RY 145 dye. The selected conditions were applied using different categories of reactive dyes as shown in Table 1.

### Effect of TNA concentration

**Figure 4:**
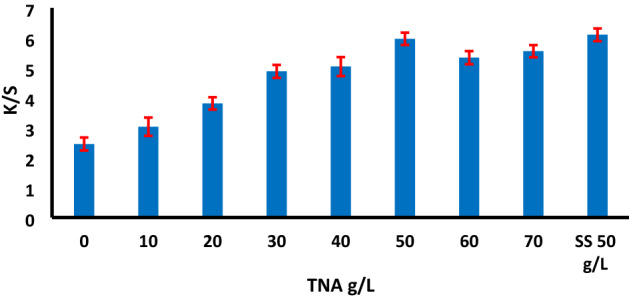
The effect of TNA concentration in the color strength (K/S) recorded on washed dyed samples for reactive dye RY 145, 2% owf, LR 1:40 at 70 °C using 50 g/L TNA compared with the conventional dyeing using SS 50 g/L and SC 20 g/L.

**Figure 5:**
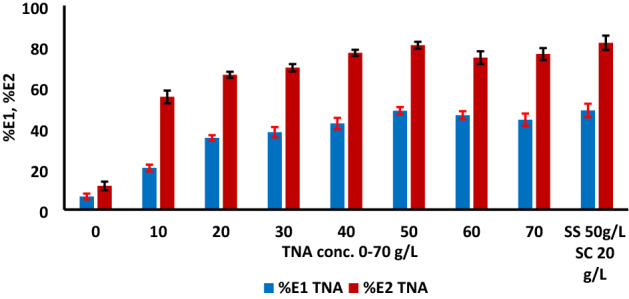
The effect of TNA concentration in primary and secondary exhaustion (%E1, %E2) for reactive dye RY 145, 2% owf, LR 1:40, %E1 40 °C after 30 min of starting dyeing, %E2 after 60 min of reach 70 °C using 50 g/L TNA compared with the conventional dyeing using SS 50 g/L and SC 20 g/L.

Studying the optimum amounts of the TNA during the dyeing process is an essential factor from the economic point of view compared with the conventionally inorganic salt (SS). Different concentrations of TNA 0–70 g/L were used for dyeing of cotton fabric with RY 145. The effect of TNA concentration on the dye exhaustion and the color strength of the dyed samples were recorded within the range 0–70 g/L used as presented in Figs. 4 and 5, respectively, from which the highest exhaustion of both of primary %E1 and secondary %E2 exhaustion, and color strength was observed with 50 g/L TNA for 2% (owf) dye shade. By comparing the conventional dyeing method with TNA method, the K/S was almost similar, with a minor increase in the case of using TNA.

Comparing to the conventional reactive dyeing method, it is believed that the degree of dye exhaustion seems to influence the extent of dye uptake and dye-fiber fixation. With the addition of TNA to the dyebath, a simultaneous reaction between the reactive dye sites and the hydroxyl groups in the fibre is formed, thus the primary exhaustion present during the initial stage of the dyeing process could lead to secondary dye exhaustion and covalent bond formation, resulting in relatively high degree of exhaustion and fixation at higher amounts of TNA, of being act as an organic base.

The data summarized in Table 2 represent the color difference ΔE*and K/S by changing TNA concentration compared with the conventionally dyed sample (reference sample). From the results, we can conclude that the ΔE* value was near to 1 at 50 g/L of TNA, which means very close in color difference compared with the blank sample.

**Table 2:** The effect of TNA concentration on the color data using 2% owf reactive dye shade of RY 145.

Concentration of TNA (g/L)	L*	a*	b*	C*	h	ΔE*	K/S 435 nm
Reference	73.95	22.56	68.09	71.73	71.66	–	6.12
0	79.69	13.70	53.43	55.15	75.62	18.07	2.32
10	78.53	16.43	58.20	60.48	74.23	12.50	3.04
20	77.72	18.75	62.32	65.08	73.26	7.88	3.85
30	76.07	20.57	65.29	68.46	72.51	4.04	4.89
40	76.15	20.85	66.20	69.41	72.52	3.37	5.07
50	75.44	22.38	68.00	71.59	71.78	1.33	5.88
60	75.75	21.20	66.51	69.81	72.32	2.76	5.21
70	75.27	22.18	67.69	71.23	71.86	1.43	5.62

Reference: The dyed sample with conventional method using 50 g/L SS and 20 g/L SC.

### Effect of sodium carbonate concentration

**Figure 6:**
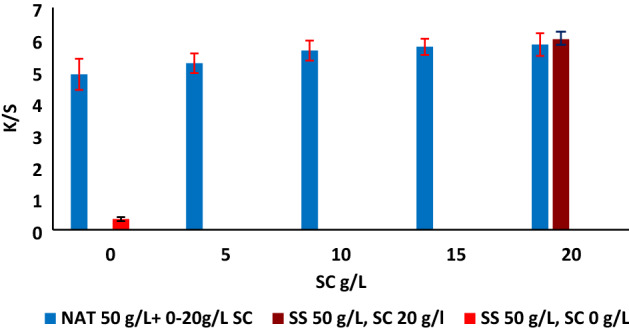
The effect of SC concentration in K/S recorded on washed dyed samples in the presence of TNA 50 g/L, 2% owf dye concentration, LR 1:40 at 70 °C, 60 min, compared with conventional dyeing using RY 145.

The effect of SC concentration on the color strength of the dyed fabrics using TNA was investigated in the presence of different SC concentrations ranging from 0 to 20 g/L and the pH of the dyebath at different concentration of SC 0, 5, 10, 15 and 20 g/L was varied from 9.5 to 11.7 in the presence of 50 g/L TNA while 0 g/L SC and 0 g/L TNA the pH was 7.5. Compared with the conventional method, the obtained K/S values revealed no significant effect in color strength and color difference, as presented in Table 3, indicating that TNA acts as a fixing agent as shown in Fig. 6. However, the presence of SC was essential for the dye fixation using SS, as its absence revealed a very low K/S value (0.33), and the color difference was very high. This very low color data is due to the unfixed dye to emphasize that the conventional method necessitates the presence of alkali for dye fixation. In other words, TNA proved to be effective as a fixing agent.

**Table 3:** The effect of sodium carbonate concentration in color data for RY 145 varied from 0 to 20 g/L while using 50 g/L TNA.

Concentration of SC (g/L)	L*	a*	b*	C*	h	ΔE*	K/S 435 nm
Reference	74.79	23.40	68.76	72.64	71.21	–	6.12
0	74.91	23.45	68.98	72.79	71.38	0.69	5.88
5	75.47	22.44	65.88	69.60	71.19	3.11	5.38
10	74.82	23.41	67.43	71.38	70.86	1.33	5.66
15	74.85	23.65	68.09	72.08	70.84	0.72	5.78
20	74.57	23.67	67.69	71.71	70.73	1.13	5.82
SS 50 g/L, SC 0 g/L	85.92	6.57	22.94	23.86	74.02	50.07	0.33

The reference sample represents the conventional dyeing method, and last raw present the dyed sample with SS instead of TNA and 0 g/L SC.

Reference: The dyed sample with conventional method using 50 g/L SS and 20 g/L SC.

### Effect of dyeing temperature

**Figure 7:**
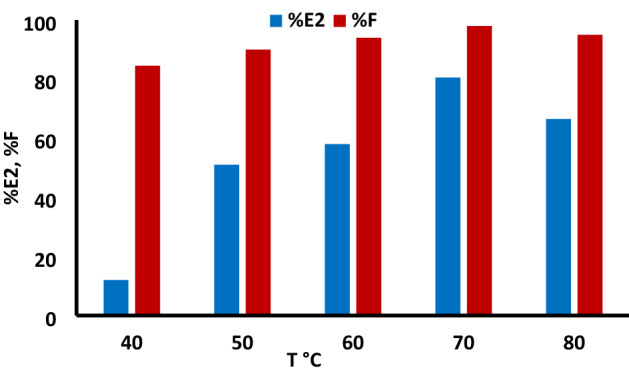
The effect of secondary exhaustion temperature using TNA 50 g/L, 2% owf of RY 145 dye, LR 1:40 at different temperatures using 50 g/L TNA for 60 min.

Secondary exhaustion and fixation was investigated at different temperatures 40–80 °C as shown in Fig. 7. The best exhaustion and fixation was observed at 70 °C for TNA method as expected for this class of dyes.

### Effect of dyeing time on primary and secondary exhaustion

**Figure 8:**
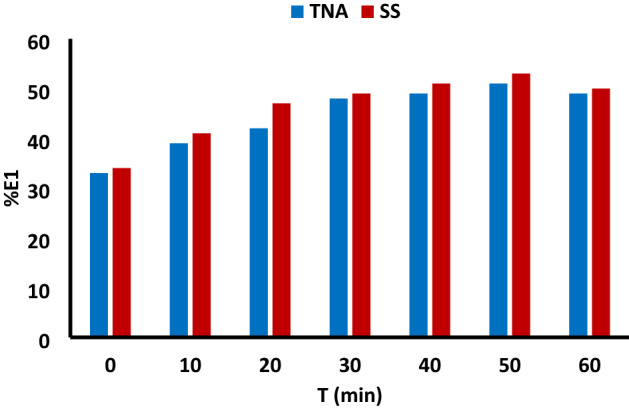
The effect of primary exhaustion time at 40 °C, 2% owf RY 145 dye concentration, LR 1:40 using 50 g/L TNA compared with conventional dyeing using 50 g/L SS, 20 g/L SC method.

**Figure 9:**
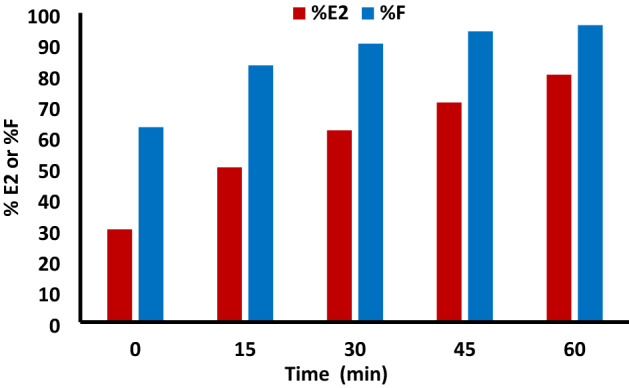
The effect of secondary exhaustion time and fixation using 50 g/L TNA, 2% owf, RY 145 dye, LR 1:40 at 70 °C.

At different times, the exhaustion was studied using both salts SS and TNA at 40 °C. As shown in Fig. 8, the primary exhaustion increased by increasing the time in both cases up to 30 min, beyond which no significant difference between both methods was observed. The effect of dyeing fixation time presented in Fig. 9 reveal an increase in the secondary exhaustion and fixation by increasing the dyeing time for 60 min at 70 °C.

**Figure 10:**
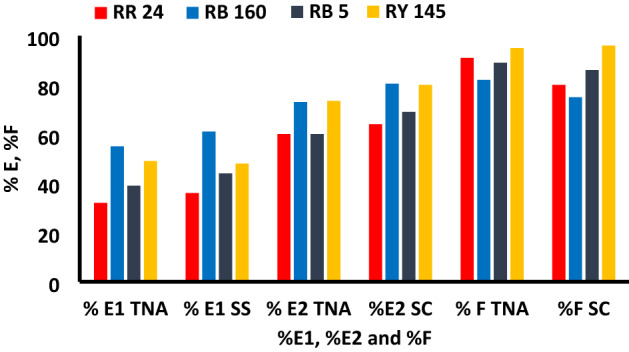
Primary (%E1), secondary (%E2) exhaustion and fixation (%F) for different reactive dyes using 50 g/L TNA, 2% owf, LR 1:40 for 60 min dyeing time at 60, 70, 80 °C for RB5, RY 145, RR 24 and RB 160, respectively and compared with conventional dyeing with 50 g/L SS and 20 g/L SC.

The dyeing behavior of different categories of reactive dyes (Table 1, Fig. 10) onto cotton fabrics was investigated to better understand the effect of using TNA instead of using a high load of inorganic salt and alkali that are conventionally used.

**Figure 11:**
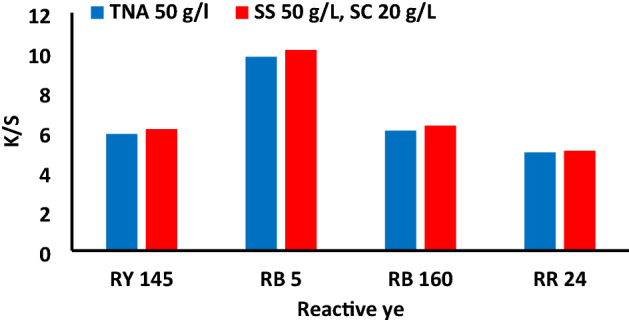
The color strength of different dyes on cotton fabric using 2%owf dye, LR 1:40 and TNA 50 g/L for exhaustion time 60 min and compared with its corresponding conventional dyeing method.

**Figure 12:**
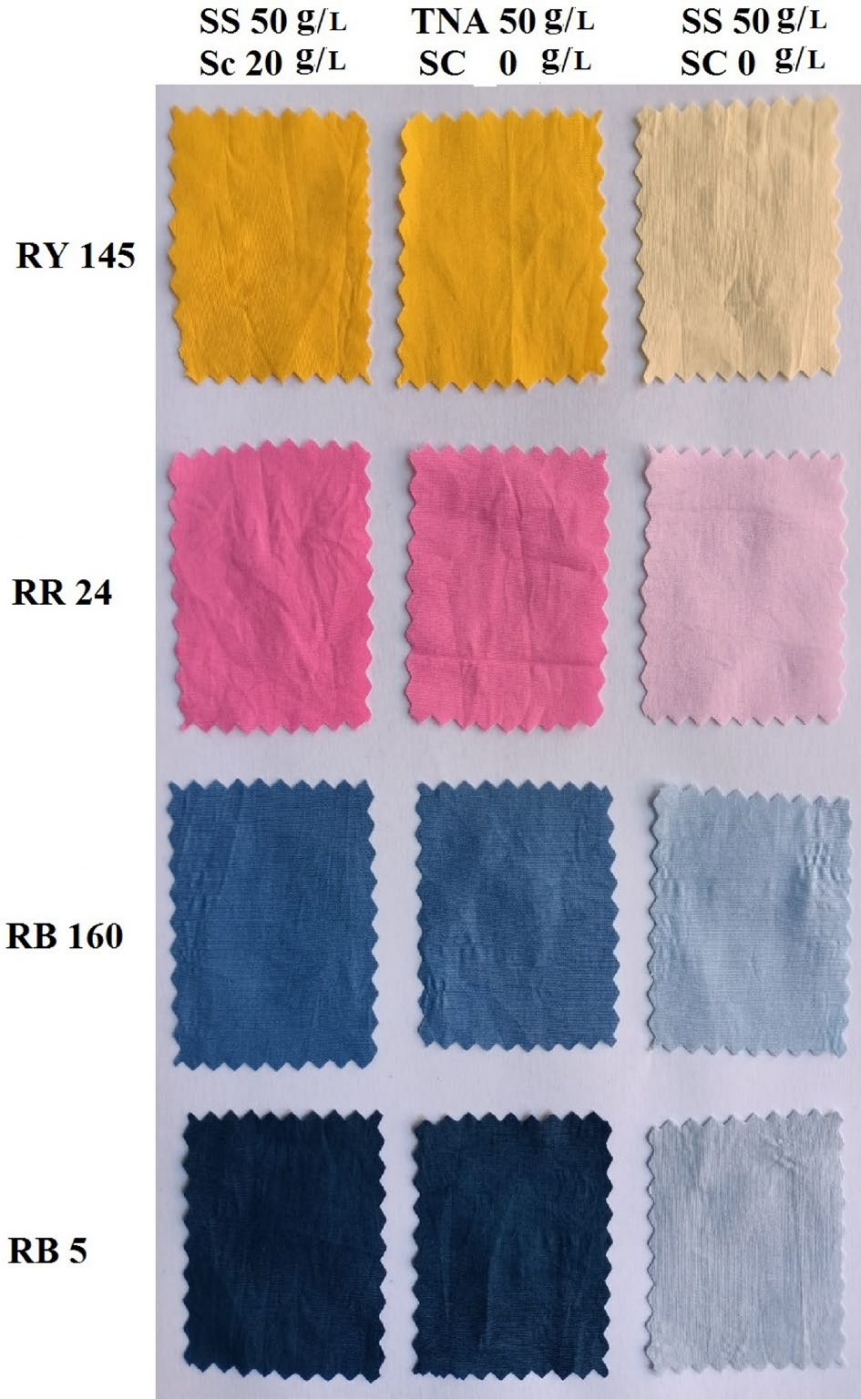
Images of the dyed cotton fabrics of different dyes using the conventional method (SS 50 g/L, SC 20 g/L compared with 50 g/L TNA methodand the dyed sample using global salt 50 g/L with 0 g/L of sodium carbonate for 2% owf, LR 1:40.

The optimum dyeing conditions using 50 g/L of TNA for 30 min at 40 °C as the primary exhaustion time and 60 min as the fixation time were applied using different reactive dyes (RB 5, RB 160, RR 24). The dye exhaustion and fixation percentages of the TNA reactive dyeing method and their color strength values are shown in Figs. 10 and 11. The results indicate that the exhaustion and fixation values were very close to those obtained using the conventional dyeing method with global salt. Images of the dyed fabrics using different dyes in the TNA method and SS method in the presence and absence of SC are shown in Fig. 12.

### Dyeing mechanism

**Figure 13:**
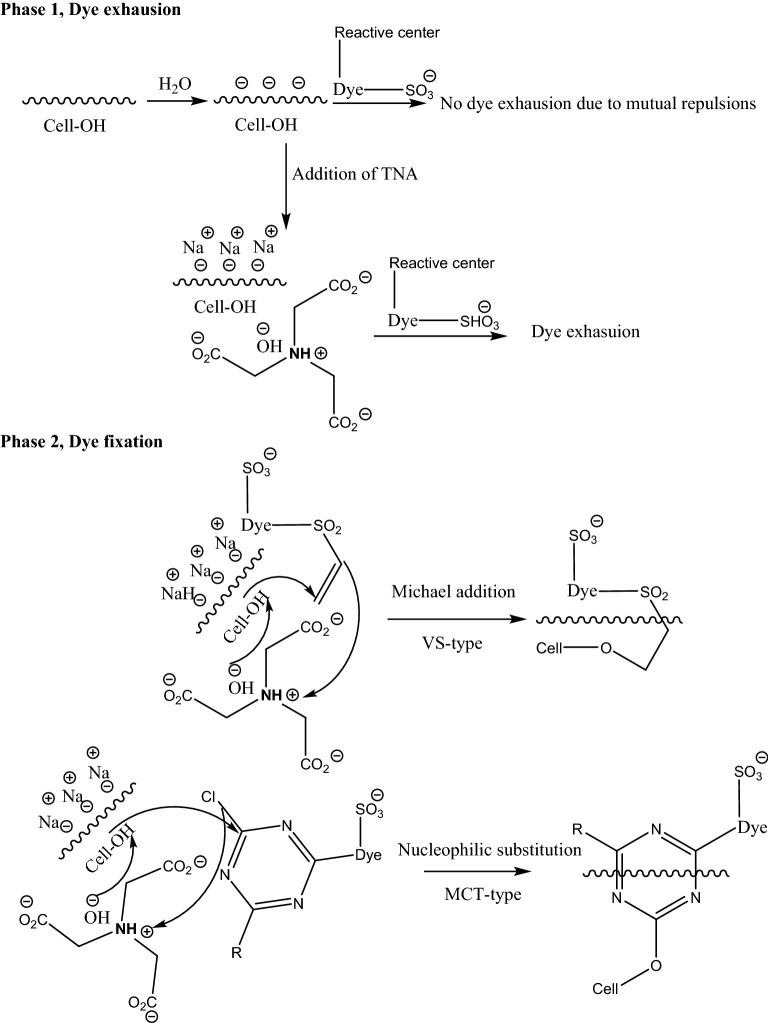
Dyeing mechanism of reactive dyes on cotton fabrics using TNA.

The use of TNA as auxiliary dyeing material for reactive dyeing of cotton fabrics proved a success as both an exhausting and fixing agent. Figure 13 shows the dyeing mechanism of reactive dyes on cotton fabric using TNA. It is known that cellulosic materials acquire negative surface charges upon dipping in water ^4^. Thus, it is conventional to add inorganic salt to cover the surface charges and avoid the mutual electrostatic repulsions between the surface of the fabrics and the dye molecules. Therefore, it was envisioned to use a biodegradable organic salt-containing amino group to act as exhausting and fixing agents. As shown in Fig. 12, the first phase of dyeing reveals the formation of an electrical double layer between the surfaces of the fabric and the sodium cations of TNA, thus suppressing the repulsion to allow dye exhaustion. In the second phase, and while the TNA molecules are in close proximity to active sites of the fabrics, proton abstraction takes place by virtue of the hydroxyl anions of TNA and thus allowing dye fixation via nucleophilic addition (VS-type reactive dyes) or nucleophilic substitution (MCT-type reactive dyes) onto cotton fabrics.

### The environmental effect of using conventional dyeing method compared with TNA method in dye effluents

The total amount of dissolved oxygen which is known as chemical oxygen demand (COD), the biological oxygen demand (BOD) and the total dissolved salt (TDS) were analyzed in both conventional and TNA effluents as summarized in Table 4. The results show a significant reduction in COD, BOD, and TDS by 99, 97, and 97%, respectively, using the TNA method compared with the conventional method.

**Table 4:** Dye effluent analysis of the dye bath after dyeing cotton fabric with RY 145 for the conventional method using SS 50 g/L, SC 20 g/L compared with 50 g/L TNA method.

Analysis	TNA method (50 g/L)	Conventional method (SS 50 g/L, SC 20 g/L)	Environmental impact using TNA (reduction %)
COD (mg/L)	269	23,871	99
BOD (mg/L)	160	6300	97
TDS (mg/L)	487	14,540	97
TSS (mg/L)	< 15	< 15	–

### Fastness properties

The results obtained using TNA method compared with those obtained using the conventional method summarized in Table 5 did not show any change in fastness properties in all studied categories of reactive dyes. These results indicate that TNA method is as effective in coloration as the conventional method and thus could be a variable alternative approach for environmentally friendly dyeing.

**Table 5:** The fastness properties of the dyed cotton fabrics (2% owf) using both conventional dyeing method (SS, 50 g/L, SC 20 g/L) and TNA method (SS/SC-free 50 g/L TNA).

Dyeing method	Reactive dyes	Rubbing		Washing			Perspiration						Light
							Acid			Alkaline			
		Dry	Wet	A	SC	SW	A	SC	SW	A	SC	SW	
TNA	RY 145	4–5	4	4	4–5	4	4–5	4–5	4–5	4–5	4–5	4	4–5
Conventional		4–5	4	4	4–5	4	4–5	4–5	4–5	4–5	4–5	4	4–5
TNA	RB 5	4–5	4	4–5	4–5	4	4–5	4–5	4–5	4–5	4–5	4–5	2–3
Conventional		4–5	4	4–5	4–5	4	4–5	4–5	4–5	4–5	4–5	4–5	2–3
TNA	RB 160	4–5	4	4–5	4–5	4	4–5	4–5	4–5	4–5	4–5	4–5	3
Conventional		4–5	4	4–5	4–5	4	4–5	4–5	4–5	4–5	4–5	4–5	3
TNA	RR 24	4–5	4	4	4–5	4	4–5	4–5	4–5	4–5	4–5	4	3–4
Conventional		4–5	4	4	4–5	4	4–5	4–5	4–5	4–5	4–5	4	3–4

*A* color change, *SC* staining on cotton, *SW* staining on wool.

## Conclusion

The potential of replacing the harmful inorganic materials loads used in the conventional batchwise reactive dyeing of cotton fabrics with TNA as an environmentally friendly alternative agent that could act by dual function as both fixing and exhausting agent has been explored. For this purpose, different classes of reactive dyes were selected to prove the success of using TNA in the dyeing exhaustion and fixation on cotton fabrics. Irrespective of the fixation mechanism (nucleophilic addition or substitution), TNA proved to be viable biodegradable auxiliary material for eco-friendly dyeing of cotton fabrics with reactive dyes. Using TNA compared with the conventional polluting method, an efficient reduction of COD, BOD, and TDS values by 99, 97, and 97%, respectively were obtained. The good reactive dyeing properties obtained suggest that TNA is a viable alternative agent for cleaner production in textile industries.

## Data Availability

All data generated or analyzed during this study are included in this published article.
